# Quality of life of young adults with non-paraphilic problematic sexual behaviors: An exploratory study

**DOI:** 10.1016/j.abrep.2018.10.003

**Published:** 2018-10-18

**Authors:** Austin W. Blum, Samuel R. Chamberlain, Jon E. Grant

**Affiliations:** a Department of Psychiatry and Behavioral Neuroscience, University of Chicago, Chicago, IL, USA; b Department of Psychiatry, University of Cambridge, UK; c Cambridge and Peterborough NHS Foundation Trust, Cambridge, UK

**Keywords:** Compulsivity, Hypersexuality, Impulsivity, Sexual behavior, Young adult

## Abstract

**Introduction:**

Many young adults are unable to control their sexual behavior despite distress or negative consequences created by these activities—a clinical phenomenon described as non-paraphilic problematic sexual behavior (PSB). Little is known about clinical features associated with quality of life in PSB.

**Methods:**

54 participants affected by PSB (ages 18–29 years) were recruited for a study on impulsivity in young adults. PSB was defined as the experience of sexual urges, fantasies, or behaviors that feel overwhelming or out of control. Participants were assessed using the Quality of Life Inventory (QOLI), other validated instruments, and questions examining aspects of health and well-being. Clinical measures associated with variation in quality of life were identified using the statistical technique of partial least squares (PLS).

**Results:**

Lower quality of life in PSB was associated with greater behavioral and self-report measures of impulsivity (specifically, Barratt attentional impulsiveness, lower age at first alcohol use), emotional dysregulation, problematic use of the internet, current suicidality, higher state anxiety and depression, and lower self-esteem.

**Conclusions:**

Impulsivity and affective problems are correlated with lower quality of life in PSB. These associations may provide a means to distinguish PSB from healthy sexual behavior.

## Highlights

• Quality of life is understudied in adults with problematic sexual behaviors.
• Lower quality of life was associated with affective problems and impulsivity.
• Higher quality of life was predicted by better self-esteem.

## 1 Introduction

Appetitive urges related to sex are common and experienced by nearly all men and women. Some people, however, experience repetitive and intense preoccupations with sexual fantasies, urges, and behaviors that result in subjective distress or psychosocial impairment (Derbyshire & Grant, 2015; Fong, Reid, & Parhami, 2012; Walton, Cantor, Bhullar, & Lykins, 2017). Although not included as a formal diagnosis in the *Diagnostic and Statistical Manual of Mental Disorders*, Fifth Edition (*DSM*-*5*; Kafka, 2014; Reid & Kafka, 2014), compulsive sexual behavior (also known as *hypersexual disorder*) has been estimated, using proposed criteria, to affect approximately 2% of young adults (Odlaug et al., 2013). The most common forms of compulsive sexual behavior include excessive masturbation, preoccupation with pornography (especially as reinforced by the internet), and ego-dystonic promiscuity, typically resulting in frequent casual sex or multiple extramarital affairs (Derbyshire & Grant, 2015). Clinically, compulsive sexual behavior is associated with high rates of psychological distress and impairment in family relationships, occupational functioning, and other areas of life (Black, Kehrberg, Flumerfelt, & Schlosser, 1997; Kuzma & Black, 2008; Spenhoff, Kruger, Hartmann, & Kobs, 2013). These problems are likely related to personality traits of impulsivity, deficits in emotional processing (alexithymia), low self-esteem, and emotional dysregulation, all of which are linked with compulsive sexual behavior (Guigliamo, 2006; Raymond, Coleman, & Miner, 2003; Reid, Carpenter, Spackman, & Willes, 2008; Reid, Stein, & Carpenter, 2011).

Compulsive sexual behavior, however, may reflect only the extreme end of a problematic sexual spectrum, and therefore the diagnosis may not capture many people who find their lives adversely affected by their sexual behaviors. For purposes of this study, we chose to examine sexual behaviors that were clinically unhealthy or problematic—defined as repetitive sexual fantasies, urges, or behaviors that are perceived to be out of control or a cause of significant distress (hereafter referred to as *non*-*paraphilic problematic sexual behavior* [PSB]; Leppink, Chamberlain, Redden, & Grant, 2016). A similar approach has been useful with other problematic behaviors, such as hazardous drinking and problem gambling, in order to assess the impact of these behaviors on clinical presentation and quality of life (Agrawal, Bucholz, & Lynskey, 2010; Carneiro et al., 2014). As in the case of these other conditions (Agrawal et al., 2010; Carneiro et al., 2014), young adults with PSB may be at heightened risk of progression to more serious forms of the behavior.

To better understand the impact of PSB on personal well-being and daily functioning, we explored the clinical correlates of quality of life associated with the behavior. Quality of life is a concept that encompasses many aspects of human experience, from the quality of our social relationships to our subjective sense of well-being. Quality of life may be especially relevant to understanding PSB given that there is no widely accepted measure of severity for the behavior—both time spent engaging in the behavior and the intensity of sexual urges or cravings, for example, are imperfect proxy indicators (Reid, 2015). Therefore, the goal of this study was to identify clinical characteristics linked with quality of life in a community sample of young adults with PSB. Because various forms of PSB have been conceptualized as addictive behaviors or as behaviors with compulsive and impulsive features (Kingston, 2015), we hypothesized that lower quality of life in PSB would be associated with poorer psychological well-being and deficits in self-control.

## 2 Methods

### 2.1 Participants

A community sample of non-treatment-seeking young adults (ages 18–29 years) meeting proposed criteria for PSB were recruited from two large metropolitan areas by media advertisements as part of an ongoing longitudinal study of impulsive behaviors. All participants underwent a detailed psychiatric evaluation (described below). Participants were excluded only if they were unable to understand or consent to the study procedures. Because we sought to examine a representative sample of young adults with PSB, and due to high rates of psychiatric comorbidity associated with sexual disorders (Derbyshire & Grant, 2015), participants with current psychiatric diagnoses were not excluded.

All study procedures were conducted in accordance with the Declaration of Helsinki. The institutional review boards of both universities involved (University of Minnesota and University of Chicago) approved the study and consent procedures. After a complete description of the study procedures, participants provided written informed consent. Participants were compensated for their time with a $50 gift card to an online retailer.

### 2.2 Assessments

#### 2.2.1 Clinical assessments

All participants underwent a semi-structured clinical interview assessing demographic characteristics, health behaviors (including diagnoses of sexually transmitted infections), and mental health issues. Participants also completed self-report impulsivity inventories and a computerized cognitive battery.

Participants were assessed for PSB using the Minnesota Impulsive Disorders Interview (MIDI), a reliable clinician-administered diagnostic instrument for disorders of impulse control, including compulsive sexual behavior (CSB; Grant, 2008). Participants were considered to have PSB if they responded “Yes” to any of the four primary diagnostic questions from the CSB module (Black et al., 1997): (1) *Do you or others that you know think that you have a problem with being preoccupied excessively with some aspect of your sexuality or being overly sexually active*? (2) *Do you have out*-*of*-*control or distressing sexual fantasies*? (3) *Do you have out*-*of*-*control or distressing sexual urges*? (4) *Do you engage in repetitive sexual behavior that you feel is out of control or causes you distress*? The MIDI was also used to screen for gambling disorder and compulsive buying. Other common psychiatric disorders were evaluated using the Mini-International Neuropsychiatric Interview (MINI; Sheehan et al., 1998). Participants at risk of suicide were identified by a non-zero cumulative score on the MINI suicidality module. In addition to the MIDI and MINI, participants were evaluated using the following instruments:

*Quality of Life Inventory* (*QOLI*; Frisch, Cornell, Villanueva, & Retzlaff, 1992). The QOLI is a self-administered measure of life satisfaction across 16 domains empirically associated with human happiness and contentment, such as health, self-esteem, money, work, love, friendships, and community. Participants rated how important each domain was to their overall happiness and satisfaction (0 = *not at all important*, 1 = *important*, 2 = *very important*) and their satisfaction in that area (−3 = *very dissatisfied* to 3 = *very satisfied*). The importance and satisfaction ratings for each domain were used to generate a weighted composite score ranging from −6 (most negative) to 6 (most positive). A total raw score reflecting the participant's overall life satisfaction was then calculated by averaging all weighted items given a nonzero importance rating. Therefore, the total raw score reflects the participant's satisfaction in only those areas of life that he or she considers important. Next, the total raw score was converted to a T-score, with cutoff scores corresponding to *high* (58–77), *average* (43–57), *low* (37–42), and *very low* (0–36) quality of life. The QOLI has demonstrated excellent reliability and validity in diverse clinical populations (Frisch et al., 1992).

*Hamilton Anxiety Rating Scale* (*HAM*-*A*; Hamilton, 1959). The HAM-A is a valid and reliable, 14-item, clinician-administered scale measuring global anxiety. The HAM-A was completed by 33 participants.

*Hamilton Depression Rating Scale* (*HAM*-*D*; Hamilton, 1960). The HAM-D is a valid and reliable, 17-item, clinician-administered scale assessing depressive symptoms. The HAM-D was completed by 48 participants.

*Rosenberg Self*-*Esteem Scale* (*RSES*; Rosenberg, 1965). The RSES is a 10-item scale measuring global feelings of self-worth or self-regard.

*Difficulties in Emotion Regulation Scale* (*DERS*; Gratz & Roemer, 2004). The DERS is a 36-item self-report measure of emotional dysregulation.

*Internet Addiction Diagnostic Questionnaire* (*IADQ*; Young, 1998). The IADQ is an 8-item self-report measure of problematic internet use. The IADQ was completed by 38 participants.

#### 2.2.2 Impulsivity questionnaires

*Barratt Impulsiveness Scale*, *Version 11* (*BIS*-*11*; Stanford et al., 2009). The BIS-11 is a 30-item self-report measure of impulsivity across attentional, motor, and non-planning dimensions.

*Eysenck Impulsiveness Questionnaire* (*I*_*7*_; Eysenck, Pearson, Easting, & Allsopp, 1985). The I_7_ is a 54-item self-report measure with three subscales: impulsiveness, venturesomeness, and empathy. For the purpose of this analysis, we examined only the impulsiveness and venturesomeness subscales.

#### 2.2.3 Cognitive assessments

Cognitive testing consisted of two previously validated paradigms from the Cambridge Neuropsychological Test Automated Battery (CANTAB; CANTABeclipse, version 3; Cambridge Cognition Ltd.). The choice of cognitive challenges was based on existing literature on cognition in PSB (Leppink et al., 2016). All testing was conducted in the same controlled environment, and the order of the tasks was fixed. Tasks were completed by 52 participants.

*Stop Signal Task* (*SST*). The SST measures control over prepotent (i.e., habitual or dominant) motor behavior. The primary outcome measure was the stop-signal reaction time (SSRT). Longer SSRTs indicate more impulsive action.

*Cambridge Gambling Task* (*CGT*). The CGT measures aspects of impulsive choice. Key outcome measures were overall proportion bet and quality of decision-making.

### 2.3 Data analysis

We identified clinical measures associated with variation in quality of life using the statistical technique of partial least squares (PLS; Garthwaite, 1994; Höskuldsson, 1988; Wold, 1966; Wold, Sjöström, & Eriksson, 2001). PLS is a versatile multivariate approach to data modeling that analyzes relationships between a response (Y) variable and any number of explanatory (X) variables by means of one or more latent factors (also known as PLS components). Unlike traditional regression, PLS simultaneously accounts for interrelationships between explanatory variables and does not assume that explanatory variables are independent (non-correlated). The Y variable was QOLI T-score and X variables were as follows: gender, age, educational level, presence of major depressive disorder, presence of clinically significant suicidality, presence of any anxiety disorder, presence of any substance use disorder, presence of antisocial personality disorder, HAM-D and HAM-A total scores, I_7_ scores (impulsiveness and venturesomeness subscales), BIS-11 scores (attentional, motor, and non-planning subscales), RSES score, DERS total score, age at first sexual activity, age of first alcohol use, history of sexually transmitted infection (STI), IADQ total score, presence of compulsive buying, presence of gambling disorder, Stop Signal Task stop-signal reaction time (SSRT), and CGT overall proportion bet and quality of decision making.

Statistical analyses were performed using JMP Pro version 13.0 (SAS Institute Inc.). Any missing data points were imputed automatically by JMP using mean substitution. The PLS model was fitted using the nonlinear iterative partial least squares (NIPALS) algorithm. We used leave-one-out cross validation and the van der Voet T^2^ test to identify the optimal model—i.e., the model with the smallest number of latent factors that did not differ significantly (*p* > 0.10) from the model with the smallest absolute predictive residual sum of squares (PRESS) value (van der Voet, 1994). We then used a two-step approach to identify the subset of predictor variables that significantly contributed to the model (i.e., explained significant variance in quality of life). First, we retained individual X variables within the model that demonstrated threshold importance by conventional criteria (variable importance for the projection [VIP] statistic >0.8; Wold, 1994). Second, in a more conservative approach, we computed 95% bootstrap confidence intervals for the standardized model coefficients of the remaining X variables and excluded those that crossed zero (*N* = 2000 bootstraps).

## 3 Results

### 3.1 Participant characteristics

Fifty-four participants with PSB (mean age = 23.6 ± *SD* 3.5 years; 67.3% male) were recruited. Most participants (70.4%) reported being excessively preoccupied with their sexuality or being overly sexually active; 31.5% reported having out-of-control sexual fantasies, 37.0% reported having out-of-control sexual urges, and 22.2% reported out-of-control sexual behaviors. The mean QOLI T-score was in the *low* range at 39.6 (11.9) [range 11–66].

The mean age of first sexual encounter was 15.3 (3.6) years. Seven participants (17.1%) had been diagnosed with an STI.

Previously diagnosed mental health problems of the sample included gambling disorder (31.5%); anxiety disorders (agoraphobia [13.0%], generalized anxiety disorder [5.6%], panic disorder [3.7%], social anxiety disorder [3.7%]); major depressive disorder (18.5%); and antisocial personality disorder (14.8%). Suicidality (i.e., current risk) was relatively common in our sample (24.1%). Current symptoms of depression and anxiety were fairly low, however, with mean scores on the HAM-A and HAM-D of 8.7 (7.4) [range 0–27] and 8.5 (7.8) [range 0–26], respectively.

On the BIS-11, mean subscale scores for participants were significantly greater than normative means: attentional = 18.3 ± *SD* 3.5 (normative mean, 16.7 ± *SD* 4.1), *t* = 2.761, *p* = 0.006; motor = 25.7 ± *SD* 4.2 (normative mean, 22.0 ± *SD* 4.0), *t* = 6.657, *p* < 0.001; non-planning = 25.8 ± *SD* 4.6 (normative mean, 23.6 ± *SD* 4.9), *t* = 3.295, *p* = 0.001. On the I_7_, the mean impulsiveness score (10.1 ± *SD* 4.5) was significantly greater than the normative mean (8.6 ± *SD* 4.4), *t* = 2.473, *p* = 0.014.

### 3.2 PLS model results

**Fig. 1:**
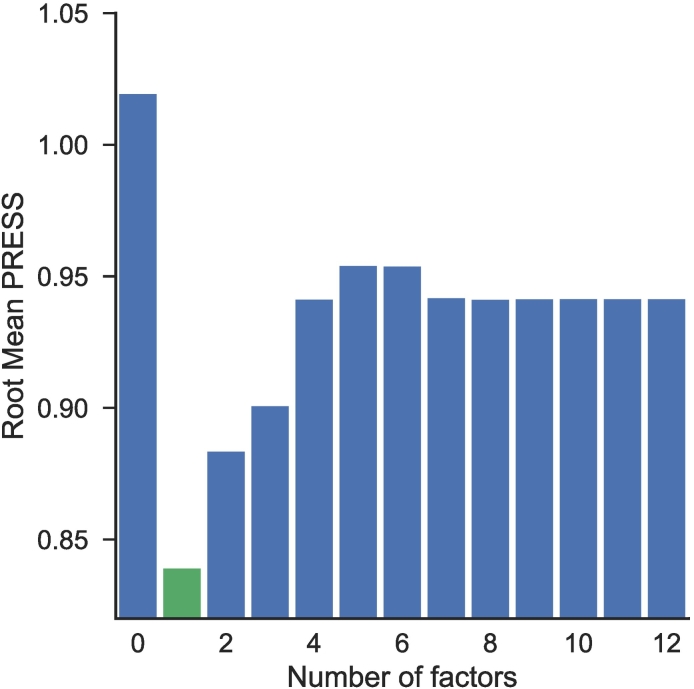
Predictive residual sum of squares (PRESS) as a function of the number of PLS latent factors. A one-factor solution (green color) provided the best fit. (For interpretation of the references to color in this figure legend, the reader is referred to the web version of this article.)

PLS identified an optimal one-factor model, which minimized the PRESS statistic (0.839; van der Voet significance level = 1.0). PLS models with two or more factors had a PRESS value of at least 0.883, as can be seen in the root mean PRESS plot (Fig. 1).

**Fig. 2:**
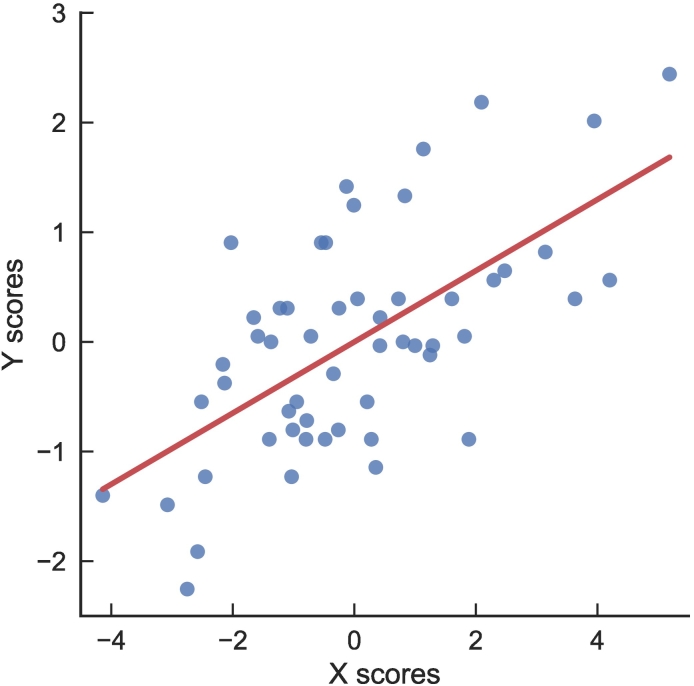
Relationship between X and Y model scores across participants in the study, showing good model fit.

The fitted model was good based on the strong relationship between X and Y scores (Fig. 2). The model explained 31.7% of the variation in the X variables and 40.1% of the variation in quality of life (Y).

Explanatory variables in the model are shown in Table 1, along with standardized model coefficients. In bootstrap, the following variables were associated with lower quality of life: state symptoms of depression and anxiety, emotional dysregulation, problematic use of the internet, BIS-11 attentional impulsiveness, and suicidality risk. In addition, two factors were protective: higher self-esteem and older age at first alcohol use.

**Table 1:** Standardized model coefficients for X variables in the optimal PLS model (one latent variable)^a^.

Variable	Coefficient
Rosenberg Self-Esteem Scale	0.1628⁎
Age at first alcohol use	0.1058⁎
Age of first sexual intercourse	0.0587
Eysenck Impulsiveness Questionnaire (I_7_)	−0.0693
Current suicidality^b^	−0.0708⁎
Barratt Impulsiveness Scale (BIS-11)–Motor Impulsiveness	−0.0738
BIS-11–Non-planning Impulsiveness	−0.0739
BIS-11–Attentional Impulsiveness	−0.0773⁎
Hamilton Anxiety Rating Scale (HAM-A)	−0.0924⁎
Internet Addiction Diagnostic Questionnaire	−0.1048⁎
Difficulties in Emotion Regulation Scale	−0.1067⁎
Hamilton Depression Rating Scale (HAM-D)	−0.1156⁎

Note. Variables with positive coefficients had a positive relationship with QOLI T-scores, and vice versa.

a Selected on the basis of variable importance for the projection (VIP) statistic >0.8.

b Defined by a non-zero cumulative score on the Mini-International Neuropsychiatric Inventory (MINI) suicidality module.

⁎ Statistically significant predictive variable by bootstrap (2000 iterations).

## 4 Discussion

To our knowledge, this is the most detailed study of quality of life in young adults affected by PSB. Using the statistical technique of PLS, we found that the covariance between quality of life and other clinical characteristics in our sample was best explained by a single latent factor. Lower quality of life in PSB was significantly and positively associated with emotional dysregulation, suicidality, problematic use of the internet, lower self-esteem, and elevated state (i.e., situational) symptoms of anxiety and depression. Aspects of impulsivity (specifically, attentional impulsiveness on the BIS-11 and lower age at first alcohol use) were also significantly associated with lower quality of life. These findings may have implications for the health and well-being of people with PSB.

Notably, we found that lower quality of life was associated with a specific measure of impulsivity: attentional impulsiveness on the BIS-11. Attentional impulsiveness is defined as the inability to concentrate or focus attention on a given task (for example, “I don't ‘pay attention’” [Stanford et al., 2009]). Other evidence implicating impaired attention in PSB comes from studies of compulsive sexual behavior (hypersexuality). Approximately 23%–27% of hypersexual men meet diagnostic criteria for attention-deficit/hyperactivity disorder (ADHD)—arguably the archetypal disorder of impulsivity—with the overwhelming majority meeting criteria for the inattentive subtype (Reid, 2007; Reid, Carpenter, Gilliland, & Karim, 2011). Hypersexual behavior (in men) has also been linked with proneness to boredom (Chaney & Blalock, 2006), a personality trait closely related to attentional impulsivity. Furthermore, heightened attentional impulsivity may be linked to emotional dysregulation in PSB, reflected by attempts to use sex to cope with stress or negative affect. Such a hypothesis is consistent with psychological studies showing that people often find it difficult to exert self-control in times of emotional distress, when immediate affect regulation is prioritized over long-term goals (Tice, Bratslavsky, & Baumeister, 2001). Thus, our results suggest that impulsivity could give rise to a range of problems affecting quality of life in people with PSB.

Although attentional impulsivity was associated with lower quality of life, other self-control processes previously implicated in PSB—including motor response inhibition (Leppink et al., 2016)—did not show such an association. Therefore, our analysis suggests that attentional problems may be more clinically relevant than deficits in other impulsivity constructs. More generally, these divergent findings illustrate the importance of fractionating impulsivity into its constituent domains. It is also worth noting one particular area requiring further study: whether impulsivity plays a global role in forms of PSB, or if it is expressed only in domain-specific contexts (such as in response to sexual stimuli; Reid, Berlin, & Kingston, 2015).

Our study also found a link between poor quality of life in PSB and problematic use of the internet. For some people, excessive or compulsive use of the internet—especially for purposes of sexual gratification—may lead to shame about the behavior (resulting in loss of self-esteem), relationship difficulties, or workplace problems (including loss of employment), with clear negative consequences for one's quality of life (Griffiths, 2012). Alternatively, online sexual behaviors may provide a short-term escape from various problems contributing to poor quality of life (Griffiths, 2012).

Consistent with previous studies, poor quality of life in PSB was associated with several emotional or psychological problems. One parsimonious explanation for these findings is that PSB and emotional difficulties may share a common antecedent: a lack of appropriate emotional regulation. From this perspective, inappropriate or excessive sexual behavior could be characterized as a maladaptive coping strategy for stress or dysphoric moods (e.g., anxiety, depression; see Black et al., 1997; Coleman, 1992; Raymond et al., 2003; Reid et al., 2008). Several findings from our study support this characterization, particularly the strong, negative association between emotional dysregulation (as measured by the DERS) and quality of life. One possibility is that people who struggle to regulate their emotions are prone to stress and rumination (Reid et al., 2008; Reid, Bramen, Anderson, & Cohen, 2014; Reid, Temko, Moghaddam, & Fong, 2014), which may make them more vulnerable to depression or anxiety interfering with quality of life. In response to these negative emotions, some people may use sex as a compensatory behavior. Some people, in fact, show paradoxically increased sexual desire and behavior when depressed or anxious, and this association appears to be especially robust in forms of disordered sexual behavior (Bancroft & Vukadinovic, 2004; Lykins, Janssen, & Graham, 2006). These behaviors offer only temporary relief from negative emotions, however, and problems resulting from PSB (such as shame [Reid, 2010; Reid, Harper, & Anderson, 2009]) may invite even more maladaptive sexual behavior in a misguided attempt to manage worsening distress. Taken together, these findings suggest that therapy focusing on cognition and emotion (i.e., cognitive–behavioral therapy and/or dialectical behavior therapy) may improve psychological well-being (and therefore quality of life) in people affected by PSB.

The present study has several limitations. Our sample included only young adults, and the clinical associations identified here may not generalize to people with PSB across a broader age range. We also note three limitations related to our clinical assessments. First, as in other studies, our analysis did not include a dimensional measure of clinical severity, as it is currently unclear how severity in PSB should be defined and measured (Reid, 2015). Second, the QOLI is a self-report assessment and may therefore under- or over-report difficulties with various life domains. Third, the BIS-11 was not specifically adapted for PSB. As noted by a previous study, using an alternative factor structure of the BIS-11 may permit a more disorder-specific assessment of impulsivity in certain clinical populations, including those affected by PSB (Reid, Cyders, Moghaddam, & Fong, 2014). Even so, we elected to use the traditional factor structure given the high rates of psychiatric comorbidity in our sample. In terms of data analysis, our use of bootstrap methods to identify statistically significant measures in the PLS model was quite conservative and may have resulted in some variables being overlooked (false negatives). Our approach does, however, provide a high degree of statistical confidence in the significant results. In addition, this study used a cross-sectional analysis and therefore cannot establish causal relationships between sexual behavior, quality of life, and other clinical variables. Despite this limitation, our analysis provides robust measures of association. Finally, the proportion of variance explained by the model was relatively modest, and other unmeasured variables are likely to be important. Future studies may wish to consider other risk factors for hypersexual behavior, such as loneliness, interpersonal sensitivity (Reid, Bramen, et al., 2014), or trauma (Howard, 2007). Sex hormone levels are also known to influence sexual behavior, though we are aware of no controlled studies examining hormonal factors in hypersexuality (Kaplan & Krueger, 2010). How these factors may influence quality of life merits further investigation.

To our knowledge, the present study is the only to examine quality of life in young adults with PSB. We found that low quality of life in PSB was associated with selective deficits in self-control—specifically, in attention and emotional regulation. Our findings therefore support the hypothesis that loss of control over sex may have pronounced effects on psychological well-being and quality of life, even among people not meeting all proposed diagnostic criteria for compulsive sexual behavior. These findings may have implications for our understanding and treatment of sexual behaviors that affect quality of life.

## Declaration of interest

Dr. Grant has received research grant support from the American Foundation for Suicide Prevention, Takeda Pharmaceuticals, and the TLC Foundation for Body-Focused Repetitive Behaviors; he receives yearly compensation from Springer Publishing for acting as Editor-in-Chief of the *Journal of Gambling Studies*; and he has received royalties from American Psychiatric Publishing, McGraw Hill, Norton Press, Johns Hopkins University Press, and Oxford University Press. Dr. Chamberlain's involvement in this project was funded by a Wellcome Trust Clinical Fellowship (reference 110049/Z/15/Z). Dr. Chamberlain serves as a consultant for Cambridge Cognition and Shire. Dr. Blum reports no financial or other potential conflicts of interest.
